# Adjusting laser power to control the heat generated by nanoparticles at the site of a patient's cells

**DOI:** 10.1049/syb2.12093

**Published:** 2024-05-24

**Authors:** Seyed Ehsan Razavi, Hamed Khodadadi, Masoud Goharimanesh

**Affiliations:** ^1^ Department of Electrical Engineering, Mashhad Branch Islamic Azad University Mashhad Iran; ^2^ Department of Electrical Engineering, Khomeinishahr Branch Islamic Azad University Isfahan Iran; ^3^ Department of Mechanical Engineering University of Torbat Heydarieh Torbat Heydarieh Iran

**Keywords:** biocontrol, medical control systems, neurocontrollers, optimisation, radial basis function networks

## Abstract

Cancer treatment often involves heat therapy, commonly administered alongside chemotherapy and radiation therapy. The authors address the challenges posed by heat treatment methods and introduce effective control techniques. These approaches enable the precise adjustment of laser radiation over time, ensuring the tumour's core temperature attains an acceptable level with a well‐defined transient response. In these control strategies, the input is the actual tumour temperature compared to the desired value, while the output governs laser radiation power. Efficient control methods are explored for regulating tumour temperature in the presence of nanoparticles and laser radiation, validated through simulations on a relevant physiological model. Initially, a Proportional‐Integral‐Derivative (PID) controller serves as the foundational compensator. The PID controller parameters are optimised using a combination of trial and error and the Imperialist Competitive Algorithm (ICA). ICA, known for its swift convergence and reduced computational complexity, proves instrumental in parameter determination. Furthermore, an intelligent controller based on an artificial neural network is integrated with the PID controller and compared against alternative methods. Simulation results underscore the efficacy of the combined neural network‐PID controller in achieving precise temperature control. This comprehensive study illuminates promising avenues for enhancing heat therapy's effectiveness in cancer treatment.

## Graphical abstract

The authors address challenges in cancer treatment involving heat therapy and introduce effective control techniques to precisely adjust laser radiation, ensuring the tumour's core temperature reaches an acceptable level with a defined transient response. The control strategies utilise an optimised Proportional‐Integral‐Derivative controller and an artificial neural network, demonstrating their efficacy in achieving precise temperature control during heat therapy and offering potential improvements in cancer treatment effectiveness.

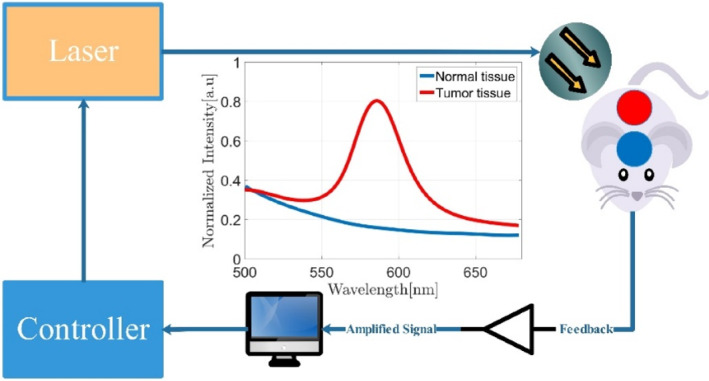

## 1 INTRODUCTION

In the United States, cancer is the second most common cause of death and the third cause of mortality globally [1, 2, 3]. Unfortunately, this issue becomes even more important when a Corona pandemic spreads worldwide, and Covid's involvement with cancer complicates the disease [4]. Recently, new methods have been developed to prevent, treat [5], and diagnose cancer [6, 7, 8, 9]. Efforts are also made to improve the efficiency of cancer treatment and reduce its complications. Thermotherapy is a novel approach that has enhanced cancer therapy. Heat treatment is often combined with chemotherapy for cancer treatment as a complementary method [10].

The drugs used in chemotherapy destroy cells with a high growth rate. Therefore, these drugs affect fast‐growing hair cells and they destroy tumour cells [3, 11]. However, these chemical drugs do not affect the dormant tumour cells and no longer proliferate. Evidence suggests that heat therapy increases the efficiency of chemotherapy from 30% to 70% [12]. The goal of the heat treatment process is to increase the temperature of the tissue (including the tumour) to a constant temperature within the range of 42–45°C and keep the temperature uniform during the treatment (20–30 min) [13]. Cells disappear at higher temperatures than 42°C, and increased temperature reduces the time it takes to kill cells. Since temperature might also affect healthy cells in the regions exposed to heat treatment, the temperature range of 43–44°C is often considered for thermotherapy [14].

Thermotherapy is based on techniques such as the induction of radiofrequency, infrared, and ultrasound waves [15]. When energy is released in heat therapy, factors known as photothermal agents, which are capable of light absorption, cause the local warming of the cancerous tissue. When photothermal agents absorb light, the electrons travel from the base to the excited state. The excitation energy generated by the excitation of the electrons is further released through non‐radiant transitions and the transfer of the kinetic energy to the surrounding environment and photothermal agents, which warm the atmosphere. Some important photothermal agents are dyes like the natural chromophores in tissues or chemicals such as nefluosianin and porphyrins, which are intermediate metals [13].

Natural chromophores are not regarded as significant photothermal agents due to their low absorption coefficient [16]. Other pigmentary materials are also necessary, especially light‐induced radiation instability [13]. Therefore, nanotechnology is utilised to overcome the limitations of photothermal agents [17]. The nanoparticles receive an external light source (e.g. a laser), turning it on with its emission. If nanoparticles operate at infrared wavelengths, they could also be applied as internal heating sources [18]. In general, the amount of generated heat depends on the laser power, wavelength, and the design of nanoparticles [19]. Notably, high‐energy external sources are not required in this technique.

Quantum dots (QDs) are nanoparticles with unique optical properties and the ability to radiate light in different colours [20, 21]. The most notable features of QDs are a broad absorption continuum, adjustable size, narrow dispatch continuum, composition utilisation ready to optimise manipulation, high shine, low light consistency, and high adjustability with living tissues [22].

In parallel to their advantages, nanoparticles have the major limitation of causing a significant increase in tissue temperature during the light exposure of external sources, such as a laser. Therefore, specific laser power is required in this method since, by increasing the laser power, the heat produced by the nanoparticles (i.e. QDs) rises and leads to the saturation of the temperature of the site. This increases the temperature of the tumour so that the temperature of the healthy tissues can be maintained for a short period. Therefore, controlling the temperature in the presence of nanoparticles is paramount since high temperatures are hazardous to healthy cells and may cause cell damage [23]. By adjusting the laser power, it would be possible to increase the temperature of the tumour tissue while also preserving healthy tissues. The present study proposed using closed‐loop control methods in a hyperthermia process.

## 2 DETERMINING THE SIZE OF NANOPARTICLES

In this stage, a range is selected for the size of Nanoparticles so that the radiation of a laser wave range is a fixed wavelength of 700 nm, and the wavelength of Nanoparticles is located in the Near‐Infrared (NIR) range. According to research results [24, 25], the enlargement of the Nanoparticles' radius results in a red‐shift emission fluoresce. Conversely, reducing the Nanoparticles' radius results in a blue‐shift emission fluoresce [26].

**FIGURE 1:**
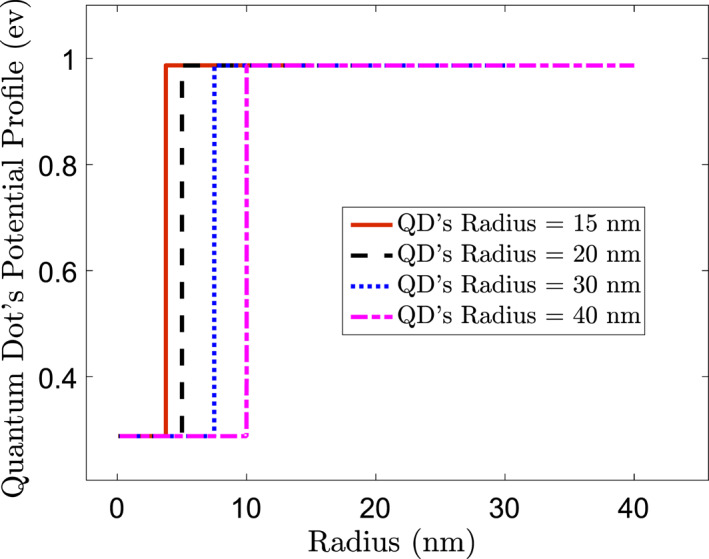
QDs potential profile (ev) versus Radius (nm). QDs, quantum dots.

**FIGURE 2:**
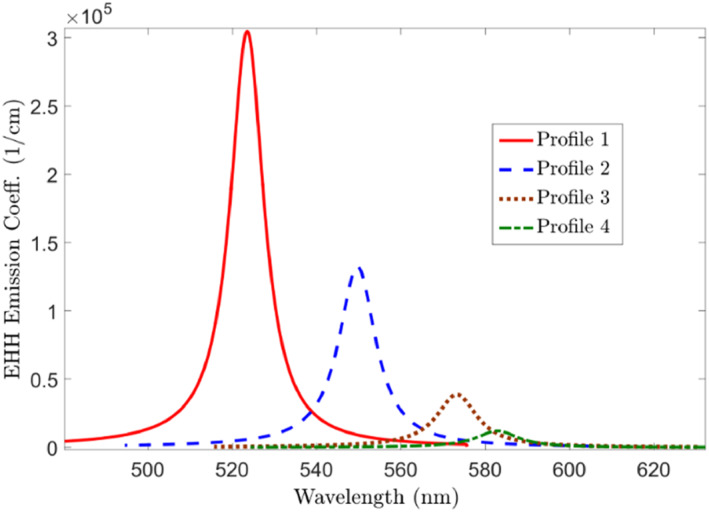
QDs emission coefficient (cm^−1^) versus wavelength. QDs, quantum dots.

Thus, the nanoparticles' radius progressively increases from zero and upwards in this simulation stage. The radiated wavelength is observed and investigated in choosing a different radius for the Nanoparticles. Finally, it is observed that if the radius of the Nanoparticles is set between 15 and 40 nm, the NIR wavelength radiates. Figures 1 and 2 show a sample of the results achieved. This figure indicates that with the increase of the QDs' radius, the emission fluoresces a red shift while the QDs' magnitude drastically decreases.

## 3 METHODOLOGY

**FIGURE 3:**
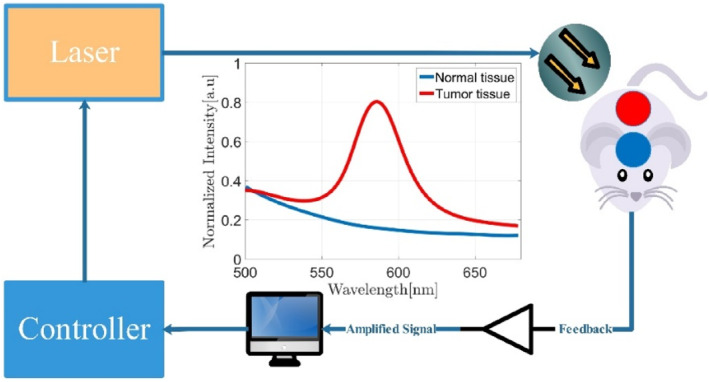
Preparation of the temperature profile from the target location with quantum dots.

Similar studies have been carried out in this field to design and simulate nanosensors and provide accurate profiles of the thermal environment or the effects of nanoparticles on the heat produced by foreign sources [27]. Our study aimed to provide a control method to control this situation so that changes in the temperature of the patient's virtual cell environment would follow a specific profile. Due to the system's speed and the temperature's performance, the controller should have a fast performance. Therefore, the main objective of the current research was to control the temperature of the environment accurately. We intended to increase the temperature of the tumour tissue by adjusting the laser power to provide the necessary conditions for tumour destruction while also maintaining healthy tissues. The designed controller must have settling time and overshoot properties, and the steady‐state error is another important parameter. Figure 3 depicts the structure of the control system in the form of a block diagram [28].

## 4 MATHEMATICAL MODEL OF THE VIRTUAL TISSUE

There have been many mathematical models proposed for thermal therapy, but the Pennes thermal equation is thought to be the best one [29].

(1) $$\rho c\frac{\partial T}{\partial t}=\nabla \left(k\nabla T\right)-w_{b}c_{b}\left(T-T_{b}\right)+Q$$

In the equation above, *c*(*J*/*kg* ^∘^ *C*) is the specific heat, *c*_*b*_ shows the specific heat of the tissue blood, *W*_*b*_(kg/m^3^sec) is the blood circulation rate, *t*_*b*_ shows the blood temperature (37°C), *Q* is the energy release function in the tissue, *k* represents the heat conduction rate, and *ρ*(kg/m^3^) is tissue density [30].

In this study, the values ​​of the parameters above were considered constant, and the researchers extracted the mean values ​​of these parameters through numerous experiments (Table 1). Concerning controlling heat therapy and since the primary model was human‐centred, it was expected that the biological parameters of the system (e.g. blood velocity, blood temperature, and tissue density) vary depending on physical conditions in each human, even in a human with changing conditions due to the changes in the estimated values. According to a clinical study, the variation range of the variables in the distribution equation is as follows [33]:

(2) $$\begin{array}{c} 3000\leq c_{i}\leq 4500,800\leq \rho \leq 1600,36\leq T_{i}\leq 39\\ 3000\leq c\leq 4500,9\leq \omega \leq 13,0.2\leq k\leq 0.6 \end{array}$$

**TABLE 1:** Mean values of pence equation parameters [31].

Sub‐region properties	Fat	Gland	Tumor
Density (*ρ*) (kg/m^3^)	930	1050	1050
Specific heat (c) (J/kg^o^K)	2770	3770	3770
Thermal conductivity (k) (W/m^o^K)	0.28	0.48	0.48
Blood perfusion properties (GC) (W/m^3^ °C)	800	2400	48,000
Metabolism heat source (*Q*_*m*_) (W/m^3^)	400	720	Ref. [32]

As can be seen, the parameters above are uncertain.

## 5 CONTROL STRATEGIES

In this research, we utilised the proportional‐integral‐derivative (PID) controller for easy implementation and performance in most cases; the controller is widely used in industries [34]. The PID controller was considered the base controller in the current research. A PID controller has three gains: proportional (*K*_*p*_), integral (*K*_*i*_), and derivative (*K*_*d*_) [35]. Changing these three parameters can improve the system to the target points. In this paper, to determine optimal controller parameters, the Imperialism Competitive Algorithm (ICA), an optimisation strategy known for its efficacy in seeking optimal parameter sets, is employed. The ICA algorithm operates by initially defining a set of optimisation variables, followed by an iterative process aimed at identifying the best parameter configuration for the given problem. In our context, the target parameters were the coefficients of the PID controller. In addition, the efficiency of the intelligent controller was compared with that of the PID controller based on an artificial neural network [36]. To improve the ability of the controller to overcome the uncertainties associated with the system dynamics, a combination of the PID controller and a neural network was used so that the artificial neural network would adjust the PID controller parameters. Following that, the performance of various controllers was compared.

It is worth noting that the successful implementation of the ICA algorithm depends on the definition of a suitable cost function. This function serves as the metric by which the algorithm evaluates and refines potential parameter sets, ensuring convergence towards an optimal solution. The cost function is defined as Equation (3):

(3) $$f_{total}=f_{Mo}+f_{tr}+f_{ts}+f_{ess}$$

In which, *tr* for rise time, *ts* for settling time, *Mo* for maximum overshoot, and *ess* for error signal—are essential metrics in evaluating system performance. Minimising the cost function described above leads to the desired system output behaviour, consistent in both transient and steady‐state responses. Lower values for maximum overshoot, rise time, and settling time contribute to rapid system responses with acceptable variations. Additionally, a reduced steady‐state error indicates minimal deviation between the system output and the desired output. Notably, the final parameter values obtained align with the optimised PID controller coefficients (optimised *K*_*d*_, *K*_*i*_, and *K*_*p*_).

### 5.1 Implementing and evaluating the control strategies

**FIGURE 4:**
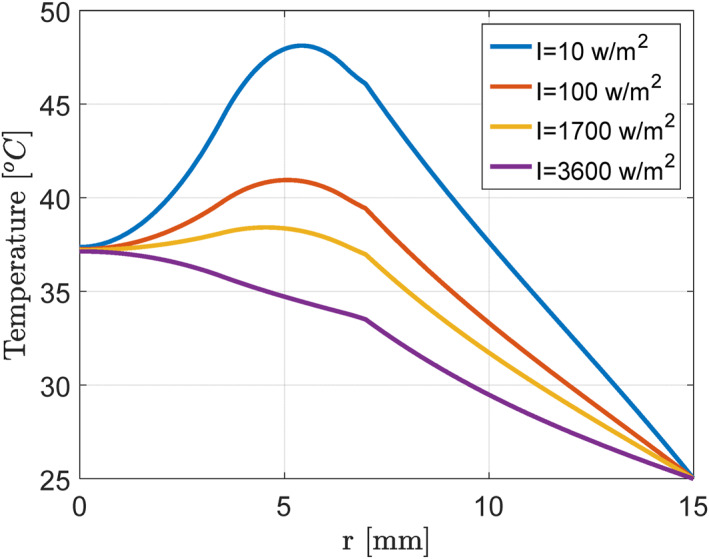
Temperature (°C) versus breast area location (mm).

**FIGURE 5:**
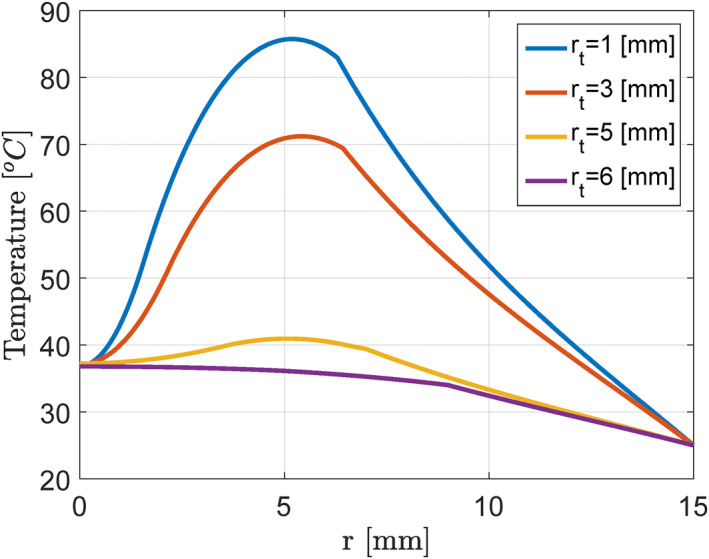
Temperature (°C) versus breast area location (mm) with an intensity of 1700 W/m^2^.

Before implementation and evaluation, a closed‐loop controller was used to determine the temperature of the tumour in the presence of nanoparticles. Initial simulation studies were designed and implemented as an open loop to emphasise the necessity of using control strategies in such a system. In other words, these studies were conducted in the absence of a closed‐loop controller. For this purpose, according to Figures 4 and 5, we considered the nanoparticle size of 15–40 nm and tumour diameter of 6 mm in the employed thermal‐critical model.

At the next stage, a virtual tumour was exposed to laser waves with a constant wavelength of 700 nm and a power of 10–3600 W/m^2^. Notably, increasing laser power from 10 to 3600 W/m^2^ sharply increased the final temperature of the tumour centre due to the presence of nanoparticles in the patient's cells. At this stage, the results showed that not having a closed‐loop controller could cause the temperature of the tumour to rise too high and the temperature of healthy tissues to rise significantly, which would cause severe damage to healthy tissues.

On the other hand, the obtained results indicated that increased tumour temperature and the subsequently high temperature of healthy tissues might occur quickly. Therefore, it is essential to design an accurate and rapid temperature controller for the texture environment so that, by adjusting the laser power, the temperature of the tumour tissue would increase without affecting healthy tissues.

### 5.2 PID controller and neural network

As mentioned in Section 3, the thermal release model is as follows [30]:

(4) $$\rho c\frac{\partial T}{\partial t}=\nabla \left(k\nabla T\right)-w_{b}c_{b}\left(T-T_{b}\right)+Q$$

Accordingly, the equations of three different tissues could be presented as follows [30]:

Gland tissue:

(5) $$\rho_{\text{Gland}}c_{\text{Gland}}\frac{{\partial T}_{\text{Gland}}}{\partial t}=K_{\text{Gland}}\nabla^{2}T_{\text{Gland}}+Q_{\text{laser}}+Q_{\text{Metabolisim}-\text{Gland}}+G_{\text{Gland}}\times C_{\text{Gland}}(T_{\text{blood}}-T_{\text{Gland}})$$

Fat tissue:

(6) $$\rho_{\mathrm{fat}}c_{\mathrm{fat}}\frac{{\partial T}_{\mathrm{fat}}}{\partial t}=K_{\mathrm{fat}}\nabla^{2}T_{\mathrm{fat}}+Q_{\mathrm{laser}}+Q_{\mathrm{Metabolisim}-\mathrm{fat}}+G_{\mathrm{fat}}\times C_{\mathrm{fat}}\left(T_{\mathrm{Gland}}-T_{\mathrm{fat}}\right)$$

Tumour tissue:

(7) $$\rho_{\text{tumour}}c_{\text{tumour}}\frac{{\partial T}_{\text{tumour}}}{\partial t}=K_{\text{tumour}}\nabla^{2}T_{\text{tumour}}+Q_{\text{laser},\mathrm{QD}}+Q_{\text{Metabolisim}‐\text{tumour}}+G_{\text{tumour}}\times C_{\text{tumour}}\left(T_{\text{Gland}}-T_{\text{tumour}}\right)$$

Six biological parameters have also been presented as problem constants, and their actual amounts vary in different individuals.

(8) $$\begin{array}{c} 3000\leq c_{i}\leq 4500,800\leq \rho \leq 1600,36\leq T_{i}\leq 39\\ 3000\leq c\leq 4500,9\leq \omega \leq 13,0.2\leq k\leq 0.6 \end{array}$$

In the equations above, *c*,*w*_*b*_,*c*_*b*_ are functions of tissue depth (tissue genus at a specific depth), *T* is the function of the depth of the texture and time, and the *k*,*ρ* values are almost constant since the exact value of parameters *ρ*, *K*, and *C* could be used to calculate the patient's image and determine the tissue around the tumor with high accuracy.

**FIGURE 6:**
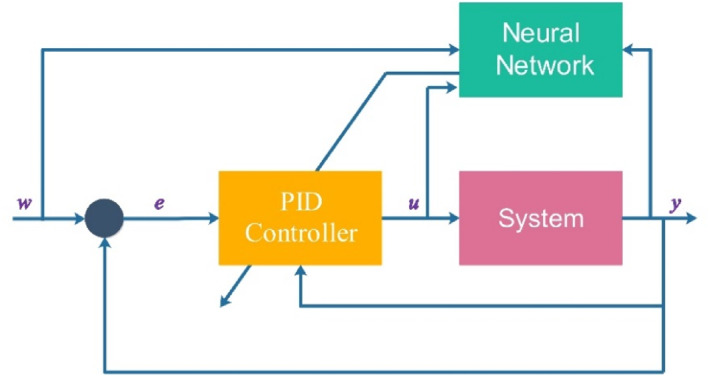
Neural network‐based PID controller. PID, Proportional‐Integral‐Derivative.

This section will discuss non‐deterministic parameters [30]. These parameters are blood‐dependent, and their precise amounts in the tumor and the surrounding tissue cannot be measured due to the continuous movement of blood. Therefore, we aimed to use a controller that could respond appropriately despite the uncertainties in the model. For this purpose, a combination of PID and neural network controllers was used (Figure 6). The ICA controller initially determined the optimal *k*_*p*_, *k*_*d*_, and ki at the defined intervals. The trained neural network [36] was then used to assess *k*_*p*_, *k*_*d*_, and *k*_*i*_ (PID controller parameters).

To enhance the controller's adaptability to uncertainties in system dynamics, a combination of the PID controller and a neural network was employed. This integration empowered the artificial neural network to adjust PID controller parameters accordingly. For the employed neural network, a hidden layer with 20 neurons applying a sigmoid activation function is designed. The output layer consist of five neurons with a linear activation function. In addition, approximately 200 data points were used for training the neural network. Besides, the Levenberg–Marquardt as a popular optimisation method was used to train the neural network. It combines aspects of gradient descent and Gauss–Newton methods to efficiently minimise the error between the actual outputs and the predicted outputs of the network.

In the present study, simulations were performed in several stages; initially, only blood perfusion (G × C) and thermal conduction were changed. As for the other parameters, their nominal values were considered.

**Step 1:** Changes in fat perfusion variables and tissue perfusion were considered within 800–1600 and 800–3600, respectively. The optimal *k*_*p*_, *k*_*d*_, and *k*_*i*_ values were also obtained from the ICA.

**FIGURE 7:**
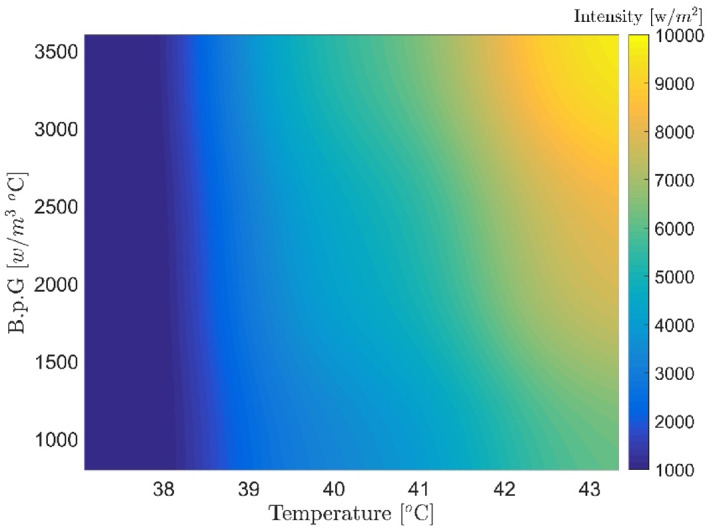
Optimised results for the proportional‐integrative‐derivative controller with ICA, Bloodperfusion_Gland = 800:400:3600 (w/m3^o^C) Bloodperfusion_fat = 1000. ICA, Imperialist Competitive Algorithm.

**FIGURE 8:**
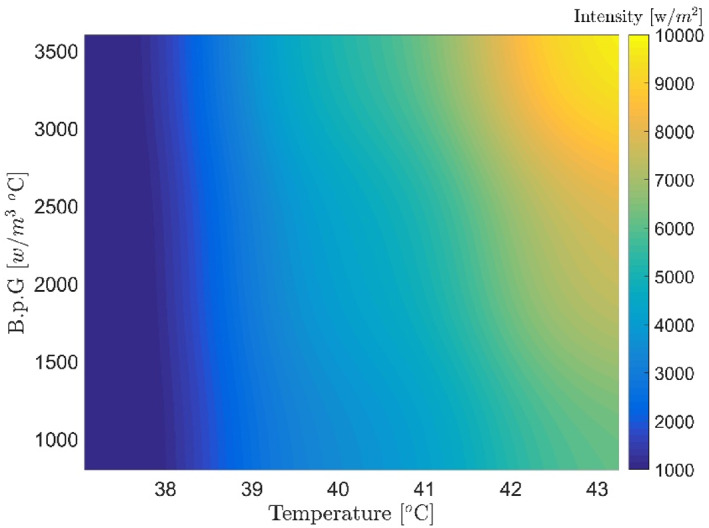
Optimised results for the proportional‐integrative‐derivative controller with ICA, Bloodperfusion_Gland = 800:400:3600 (w/m3^o^C) Bloodperfusion_fat = 800. ICA, Imperialist Competitive Algorithm.

**FIGURE 9:**
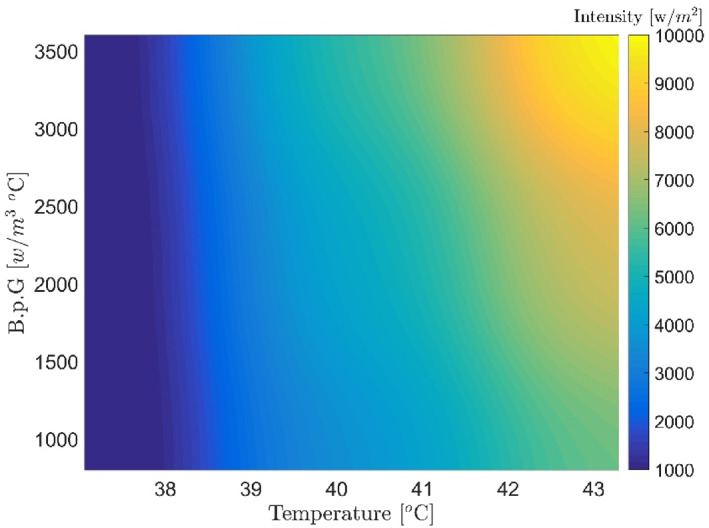
Optimised results for proportional‐integrative‐derivative controller with ICA, Bloodperfusion_Gland = 800:400:3600 (w/m3^o^C) Bloodperfusion_fat = 1400. ICA, Imperialist Competitive Algorithm.

**FIGURE 10:**
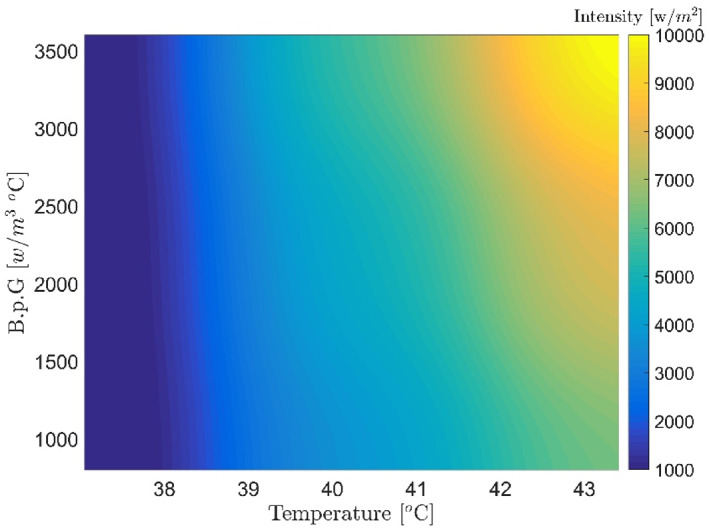
Optimised results for proportional‐integrative‐derivative controller with ICA, Bloodperfusion_Gland = 800:400:3600 (w/m3^o^C) Bloodperfusion_fat = 1200. ICA, Imperialist Competitive Algorithm.

**FIGURE 11:**
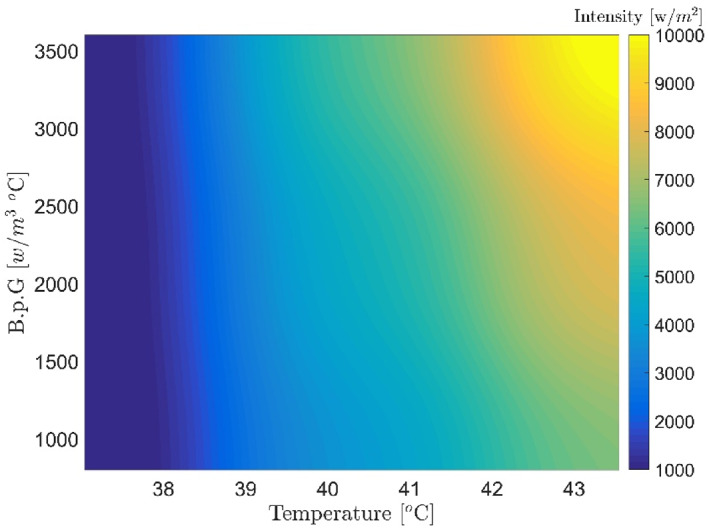
Optimised results for proportional‐integrative‐derivative controller with ICA, Bloodperfusion_Gland = 800:400:3600 (w/m3^o^C) Bloodperfusion_fat = 1600. ICA, Imperialist Competitive Algorithm.

**FIGURE 12:**
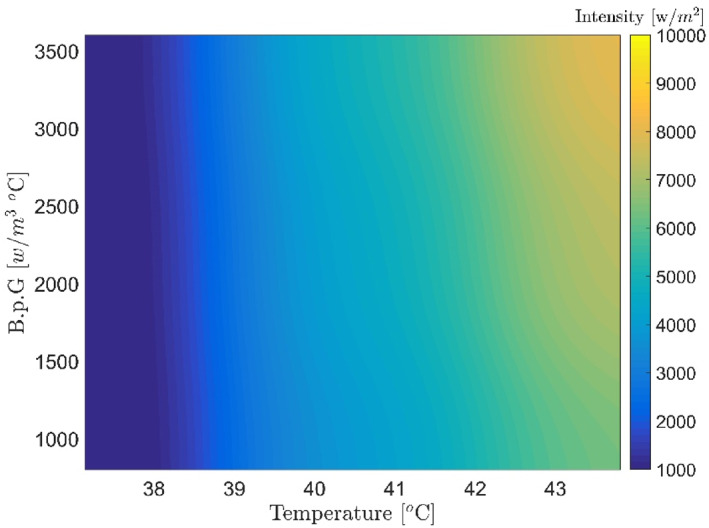
Optimised results on the PID controller with ICA, Blood perfusion gland = 800:400:3600, blood perfusion fat = 1400, thermal conductivity = 0.6. ICA, Imperialist Competitive Algorithm; PID, Proportional‐Integral‐Derivative.

Figures 7, 8, 9, 10, 11, 12 show that despite significant changes in blood perfusion, the transient response to the system did not change significantly, implying that the rise time, settling time, and overshoot did not increase significantly. Therefore, it could be concluded that significant changes in the basic parameters of the system over their nominal values did not diminish the performance of the controller. Because of the uncertainty about blood perfusion, it was also clear that a good transient response would increase the laser power density by making the blood perfusion go up.

**Step 2:** At this stage, the thermal conductivity values ​​of the variables were considered in addition to the blood perfusion changes of the tissue. To avoid repetition, a sample of the optimal results has been presented below.

The obtained results demonstrated that by changing the thermal conductivity of the tissue, the laser power density decreased with increased thermal conductivity compared to the nominal values (maximum value: 8000–10,000 W/m^2^). Despite significant blood perfusion and thermal conductivity changes, the system's transient response did not change significantly, implying that the rise and settling times did not change substantially. Along with changing the blood flow, the values of thermal conductivity and the other parameters were chosen at random, and the neural network and PID controller were tested for how well they worked (Figure 15 and Table 4).

**FIGURE 13:**
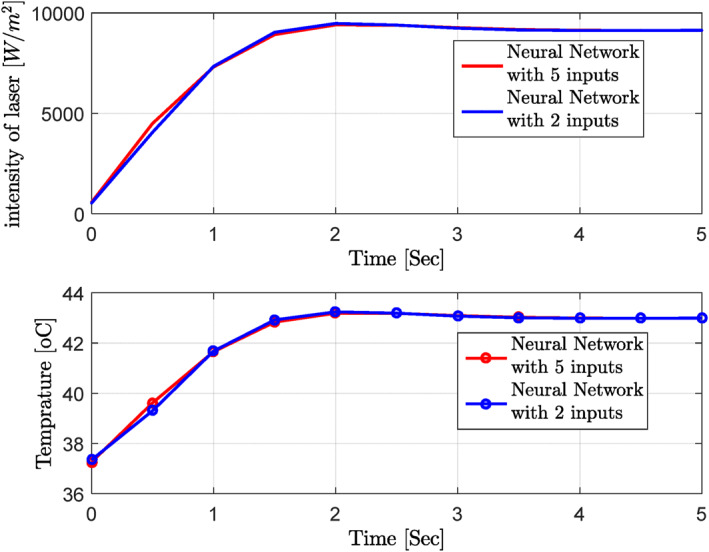
Optimised results on PID and neural network controllers (Blood perfusion gland = 850, blood perfusion fat = 3,070, thermal conductivity = 0.27, 0.51, and 0.53). PID, Proportional‐Integral‐Derivative.

**Step 3:** At this stage, the optimal results obtained from the previous steps (ICA) were used in the training of the neural network to respond to the uncertainty and changes of the parameters appropriately (Figure 13; Table 2).

**TABLE 2:** Combination of PID and neural network controllers.

Kd = 101.8		KI = 1458		Kp = 539.5377
Tr	Ts	Overshoot (%)	Error signal	Signal_end
0.5000	1.5000	0.6880	0.0893	42.9616

Abbreviation: PID, Proportional‐Integral‐Derivative.

**FIGURE 14:**
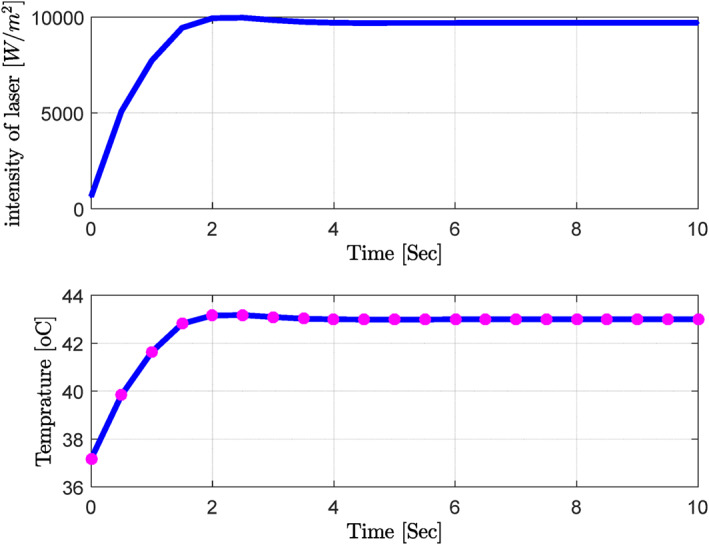
Combination of the PID controller and neural network for random changes in blood perfusion values (Blood perfusion gland = 3,357, blood perfusion fat = 901.58). PID, Proportional‐Integral‐Derivative.

At the next stage and in the presence of a trained neural network, the blood perfusion values were randomly selected, and the trained neural network was applied to extract the optimal values of PID controller parameters (Figure 14; Table 3).

**TABLE 3:** Combination of the PID controller and neural network with random changes in blood perfusion values.

Kd = 51.28		KI = 1755		Kp = 638.42
Tr	Ts	Overshoot (%)	Error signal	Signal_end
0.5000	1	0.4233	1.2571e−005	43.0000

Abbreviation: PID, Proportional‐Integral‐Derivative.

**FIGURE 15:**
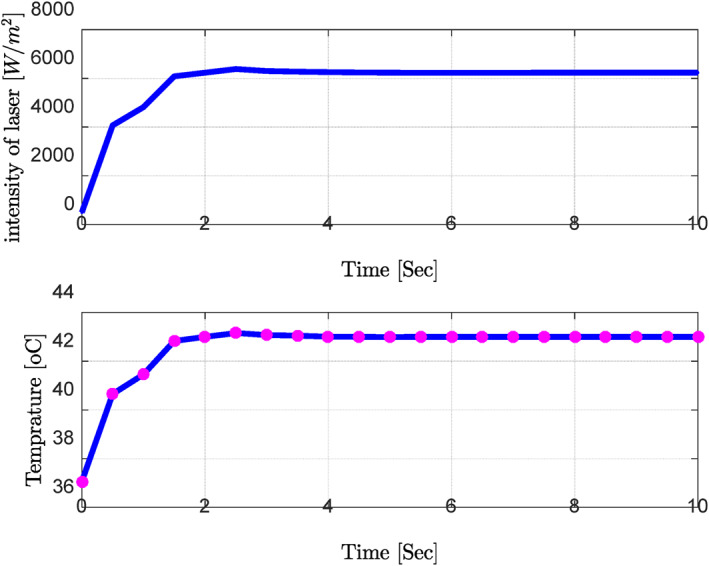
Combination of the PID controller and neural network for random changes in all parameters. PID, Proportional‐Integral‐Derivative.

According to the obtained results, combining the trained neural network with the PID controller could control the ambient temperature with a proper transient response at an acceptable steady‐state error. For instance, the rise and settling times were considerable due to the speed of dynamic system changes. Along with changing the blood flow, the values of thermal conductivity and the other parameters were chosen at random, and the neural network and PID controller were tested for how well they worked (Figure 15 and Table 4).

**TABLE 4:** Combination of the PID controller and neural network for random changes in all parameters.

Kd = 84.74		KI = 1342.5		Kp = 581.77
Tr	Ts	Overshoot (%)	Error signal	Signal_end
0.5000	0.5000	0.8080	1.9190e−004	42.9999

Abbreviation: PID, Proportional‐Integral‐Derivative.

Changes in the laser power and ambient temperature caused changes in the blood perfusion parameters, thermal conductivity, and other parameters. However, the changes in the different parameters did not have any major effects on the system.

### 5.3 Comparison

To assess the effectiveness of the proposed controller, a comprehensive comparison was conducted involving the controller proposed in this paper, a neural network‐based controller, and a PID controller optimised via ICA. The outcomes of this comparative analysis are detailed in Table 5, providing a clear insight into the performance metrics of each controller under examination. Notably, in scenarios characterised by system uncertainty, the proposed controller consistently demonstrated superior performance when compared with the other controllers considered in the study. This emphasises the robustness and efficacy of the proposed controller in handling complex and uncertain system dynamics, showcasing its potential for real‐world applications where such challenges are established.

**TABLE 5:** Comparative analysis of the proposed method and other controllers in uncertain condition.

Controller type	Tr	Ts	Overshoot (%)	Error signal	Signal_end
PID optimised by ICA	0.5000	2.5000	20.4979	11.5010	37.1696
Neural network	0.5000	2.0000	15.741	6.0423	39.2317
Proposed method	0.5000	0.5000	0.8080	1.9190e−004	42.9999

## 6 RESULTS AND DISCUSSION

The PID classic controller that its parameters are tuned based on ICA, an optimisation algorithm, is employed in this study. The ICA solutions indicated that ambient temperature control is accomplished, and the behaviour of the transient system is appropriate. An artificial neural network‐based smart controller is also evaluated in this study. This intelligent controller also controlled the ambient temperature and had an appropriate transient response. Notably, the PID controller, the parameters determined using the ICA, produced a better transient response than the neural network controller. Continued research is recommended to enhance the function of the PID controller, clarify the variable dynamics with a controlled system time, and determine the controller parameters based on a neural network.

In the present study, simulations are carried out in several stages; only blood perfusion (G × C) and thermal conductivity are changed. As for the other parameters, their nominal values are considered. Despite significant changes in blood perfusion, the transient response to the system did not change significantly, implying that the rise time, settling time, and overshoot did not have a significant increase in elevation. Therefore, it could be concluded that significant changes in the basic parameters of the system over their nominal values will not diminish the performance of the controller. The uncertainty regarding blood perfusion also indicated that an appropriate transient response would increase the laser power density by increasing blood perfusion.

In addition to changes in the blood perfusion of the tissue in the second step, the thermal conductivity values ​​of the variables are considered. By changing the thermal conductivity of the tissue, the decreased laser power density with increased thermal conductivity is compared to the results obtained from the nominal values (maximum value of 8000–10,000 W/m^2^).

At the final stage, the uncertainties associated with the model are evaluated and controlled using the combination of the neural network controllers and ICA to hold the ambient temperature. According to the findings, changes in the blood perfusion parameters, thermal conductivity, and other parameters are affected by changes in the laser power and ambient temperature, while the other parameters had no significant effects on the system.

## 7 CONCLUSION

Our findings indicated that combining the trained neural network with the PID controller could control the ambient temperature with a proper transient response at the same acceptable steady‐state error. For instance, the rise and settling times were considerable due to the speed of the system's dynamic changes. In conclusion, it could be argued that the controllers designed at the controlled ambient temperature could provide an appropriate, durable transient response even in the presence of uncertainties. By applying disturbances to the system, the environment could be properly controlled.

**Nomenclature: List of Symbols**
QD: Quantum‐Dots
QD‐QW: Quantum‐Dot Quntum‐Well
NPs: Nanoparticles
FEM: Finite Element Method
NIR: Near‐Infrared
PID: Proportional Integral Derivative
ICA: Imperialism Competitive Algorithm
NN: Neural Networks

**Symbolic Naming**
E: Total emitted radiance (W/m2)
H: Average convection coefficient (W/m2)
K: Thermal conductivity (W/m oK)
Q: Heat generation (W/m3)
T: Time (s)
S: Sum of squares
T: Temperature (°C)
X: Sensitivity coefficient matrix
Σ: Stefan‐Boltzmann constant (W/m2 K4)
GC: Blood perfusion term (W/m3 oC)
C: Specific heat (J/Kg oK)
A: absorption
S: scattering

## AUTHOR CONTRIBUTIONS

**Seyed Ehsan Razavi**: Software; Writing – original draft; Conceptualization; Supervision; Project administration. **Hamed Khodadadi**: Formal analysis; Writing – review & editing. **Masoud Goharimanesh**: Validation; Methodology.

## CONFLICT OF INTEREST STATEMENT

The authors declare that they have no known competing financial interests or personal relationships that could have appeared to influence the work reported in this paper.

## Data Availability

Data sharing not applicable—no new data generated, or the article describes entirely theoretical research.
